# Pharmacokinetic Study of Triptolide Nanocarrier in Transdermal Drug Delivery System—Combination of Experiment and Mathematical Modeling

**DOI:** 10.3390/molecules28020553

**Published:** 2023-01-05

**Authors:** Meng Yang, Jianxia Meng, Lu Han, Xiaoyan Yu, Zhimin Fan, Yongfang Yuan

**Affiliations:** 1 Department of Pharmacy, Shanghai Ninth People Hospital, Shanghai Jiao Tong University, Shanghai 200011, China; 2 College of Pharmacy, Shandong University of Traditional Chinese Medicine, Jinan 250355, China

**Keywords:** triptolide, transdermal drug delivery, nanoemulsion gel, multiscale mathematical modeling, synchronous microdialysis

## Abstract

Compared with traditional oral and injection administration, the transdermal administration of traditional Chinese medicine has distinctive characteristics and advantages, which can avoid the “first pass effect” of the liver and the destruction of the gastrointestinal tract, maintain a stable blood concentration, and prolong drug action time. However, the basic theory and technology research in transdermal drug delivery are relatively limited at present, especially regarding research on new carriers of transdermal drug delivery and pharmacokinetic studies of the skin, which has become a bottleneck of transdermal drug delivery development. Triptolide is one of the main active components of *Tripterygium wilfordii*, which displays activities against mouse models of polycystic kidney disease and pancreatic cancer but its physical properties and severe toxicity limit its therapeutic potential. Due to the previously mentioned advantages of transdermal administration, in this study, we performed a detail analysis of the pharmacokinetics of a new transdermal triptolide delivery system. Triptolide nanoemulsion gels were prepared and served as new delivery systems, and the ex vivo characteristics were described. The metabolic characteristics of the different triptolide transdermal drug delivery formulations were investigated via skin–blood synchronous microdialysis combined with LC/MS. A multiscale modeling framework, molecular dynamics and finite element modeling were adopted to simulate the transport process of triptolide in the skin and to explore the pharmacokinetics and mathematical patterns. This study shows that the three−layer model can be used for transdermal drug delivery system drug diffusion research. Therefore, it is profitable for transdermal drug delivery system design and the optimization of the dosage form. Based on the drug concentration of the in vivo microdialysis measurement technology, the diffusion coefficient of drugs in the skin can be more accurately measured, and the numerical results can be verified. Therefore, the microdialysis technique combined with mathematical modeling provides a very good platform for the further study of transdermal delivery systems. This research will provide a new technology and method for the study of the pharmacokinetics of traditional Chinese medicine transdermal drug delivery. It has important theoretical and practical significance in clarifying the metabolic transformation of percutaneous drug absorption and screening for appropriate drugs and dosage forms of transdermal drug delivery.

## 1. Introduction

Systemic administration (transcutaneous administration) has several advantages over oral administration, such as avoiding the whole body metabolism of the gastrointestinal tract and liver, having a controllable drug release rate, providing continuous plasma drug concentrations, and improving patient compliance [1]. Another advantage is that the incidence and severity of side effects can be reduced. However, a major obstacle for this route of administration is the low permeability of many drugs, which hinders the effective dose of patch administration in terms of acceptable physical sizes [2,3].

Triptolide (TPL) is the main active ingredient of *Tripterygium wilfordii*, which has anti−inflammatory, analgesic, antitumor and immune regulation properties. Oral TPL develops a highly local toxicity that seriously limits its clinical application [4]. Transdermal administration is an important method that effectively reduces these adverse reactions. However, for transdermal drug delivery, the concentration of drugs in plasma is low and is difficult to detect. The rich enzyme system in the skin also leads to many complex products during drug metabolism or other actions in transdermal transport. The serum (plasma) contains many substances that are difficult to separate and identify, limiting the in−depth study of transdermal drug delivery preparations [5]. A large number of studies have shown that nanotechnology has significant advantages in improving drug stability, promoting drug transdermal absorption, and controlling drug release and targeted drug delivery, which strongly promotes the development of transdermal drug delivery systems [6,7,8,9]. Nanocarriers not only promote the molecular mechanism of drug transdermal absorption but also affect drug metabolism and other processes. To improve drug permeability through the skin, the nanoscale carriers of lipid nanoparticles can contribute to drug delivery as their contact with the cuticle of the skin is better, increasing the contact time and contact area. In this way, adequate drug permeability through the skin can be achieved.

Microdialysis (MD) is a new type of sampling technique that reflects the metabolism in the local skin. Compared with the sampling methods of skin biopsy, suction blistering, and tape stripping, MD hardly damages the skin at all. The membrane of the MD probe intercepts proteins, enzymes, large molecules, and the dialysis solution can be further investigated without pre−treatments [10]. The advantages of MD, such as in situ online real−time detection, make it particularly suitable for use in traditional Chinese medicine (TCM) transdermal delivery system studies in vivo [11].

Mathematical studies have shown that the model of drug penetration through the skin can provide important insights for transdermal drug delivery (TDD) systems and be considered an important basis for analyzing TDD. However, in the 1960s, Takeru Higuchi first used Fick’s first diffusion law to link these characteristics with passive diffusion in drug molecular transdermal absorption and carefully attempted to establish the TDD drug diffusion model [12,13,14]. Based on diffusion law, more and more technologies have been used for simulation in drug transport research, thereby introducing TDD. 

It is hypothesized that the same skin and drug molecules can be used to obtain more realistic drug delivery behaviors and instantaneous drug concentrations by averaging the simulation results, which are given by different histological images depicting the diffusion coefficient or permeability of the drug in the corresponding situation. Molecular dynamics simulation can realize the dynamic behavior of molecules in real time and intuitively understand the evolution of the system under certain conditions to reflect the macro characteristics of the system. It is precisely molecular dynamics simulation that can show the dynamic behavior of molecules in real time without the limitation of sample preparation and experimental technology in order to more intuitively understand the changing process of the system under certain conditions [15,16].

This study used triptolide as a model drug, establishing microdialysis coupled with LC–MS/MS to study the pharmacokinetic parameters of three different formulations (triptolide nanoemulsion, nanoemulsion−based gels, and marketed triptolide ointments). To establish a multistage modeling framework, this study employed ex vivo permeation experiments to measure the drug matrix diffusion coefficient, and microdialysis experiments were used to measure the drug concentration in the skin by fitting the experimental data to obtain the diffusion coefficient of triptolide in the skin. Then, finite element modeling was used to simulate the dynamics of the three formulations in the transdermal drug delivery system, drugs in receptor cells were analyzed to obtain a drug concentration time curve, and the therapeutic effects of different dosage forms were compared. Multiscale modeling was used to simulate the diffusion and absorption process in the skin via molecular dynamics simulation and finite element analysis to explore the kinetic process and mathematical laws of the drug. This research will provide new technology and a method for the study of the pharmacokinetics of traditional Chinese medicine transdermal drug delivery. It has important theoretical and practical significance in clarifying the metabolic transformation of percutaneous drug absorption and screening for appropriate drugs and dosage forms of transdermal drug delivery.

## 2. Results

### 2.1. Pharmacokinetis Study of Triptolide

**Figure 1:**
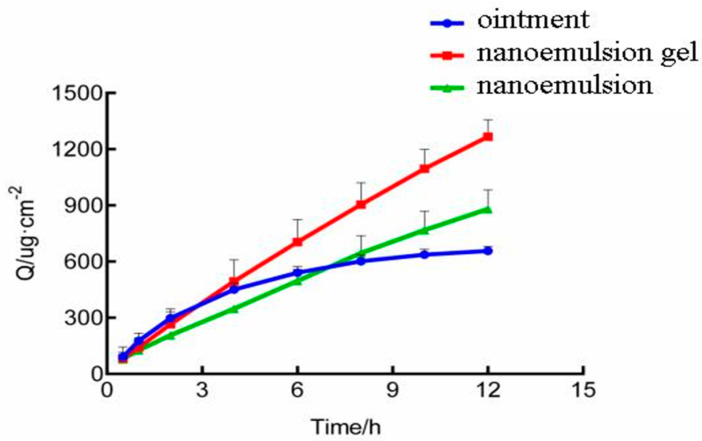
Experimental measurement data (Q is percutaneous cumulative release).

The transdermal properties were investigated using a Franz diffusion cell. The triptolide−saturated aqueous solution, triptolide nanoemulsion and triptolide micro−based gel was used to perform the experiment and the results are shown in Figure 1.

From the results, for triptolide in the three drug delivery systems, the 12 h cumulative permeation rate and penetration rate had the following order: triptolide nanoemulsion gel > triptolide nanoemulsion > triptolide ointment. This suggests that triptolide nanoemulsion gels and nanoemulsions are favorable for percutaneous absorption.

The infusion speed of the infusion fluid was chosen as 5 μL/min. The recoveries of the skin linear probe and blood vessel concentric circle probe affected by the triptolide concentration were detected with the gain method and loss method. The results shown in Table 1 indicate that the recoveries of the skin linear probe and blood vessel concentric circle probe detected with the two methods were consistent under different triptolide concentration levels. The triptolide concentration did not significantly affect the recoveries of the kinds of probes of the two types.

The in vivo recoveries of two kinds of MD probes were detected with the loss method. The results shown in Table 2 indicated that at an infusion speed of 5 μL/min, the in vivo recoveries were consistent at different triptolide concentration levels. The in vivo recovery of the linear probe was 69.7% ± 4.8%, and the in vivo recovery of the concentric circle probe was 51.6% ± 7.2%. As known the results of ex vivo recovery, the recoveries of triptolide with the gain method and loss method were basically consistent. Therefore, the triptolide concentration during the in vivo pharmacokinetics course can be calculated with corrected in vivo recovery.

(1) $$C=\frac{C\;\mathrm{dialysis}}{R\;\mathrm{in}\;\mathrm{vivo}}$$

In the methods of MD sampling, the correction of probe recovery is the critical step for the true drug concentration in tissue fluid. RR indicates the ratio of drug recovered from tissue fluid by the probe.

(2) $$\mathrm{RR}=\frac{C_{d}-C_{p}}{C_{m}-C_{p}}$$

where C_p_ = the drug concentration in the infusion solution; C_d_ = the drug concentration in the dialysis solution; and C_m_ = the drug concentration in the tissue fluid.

In the above formula, when the drug concentration in the infusion solution is lower than that in the circumambient tissue fluid, the drug diffuses from the circumambient tissue to the MD probe; here, the probe recovers the drug from the circumambient tissue, and RR is the recovery. However, when the drug concentration in the infusion solution is higher than that in the circumambient tissue fluid, the drug diffuses from the infusion solution at a higher concentration to the circumambient tissue fluid via the dialysis window of the MD probe; here, RR is the delivery of the drug by the probe. The theory of MD probe recovery correction employing the reverse dialysis method is that the recovery of the probe is equal to its delivery. When both of them are equal, it decreases the error of the test using drug delivery to correct the relative recovery, and it is a precise correction method. However, the equivalence between the relative recovery and delivery is laid on the property of the drug, and not all of the drugs are suitable. Hence, the equivalence between the relative recovery and delivery must be proven before the reverse dialysis method is employed to correct the relative recovery of the MD probe [17,18].

**Figure 2:**
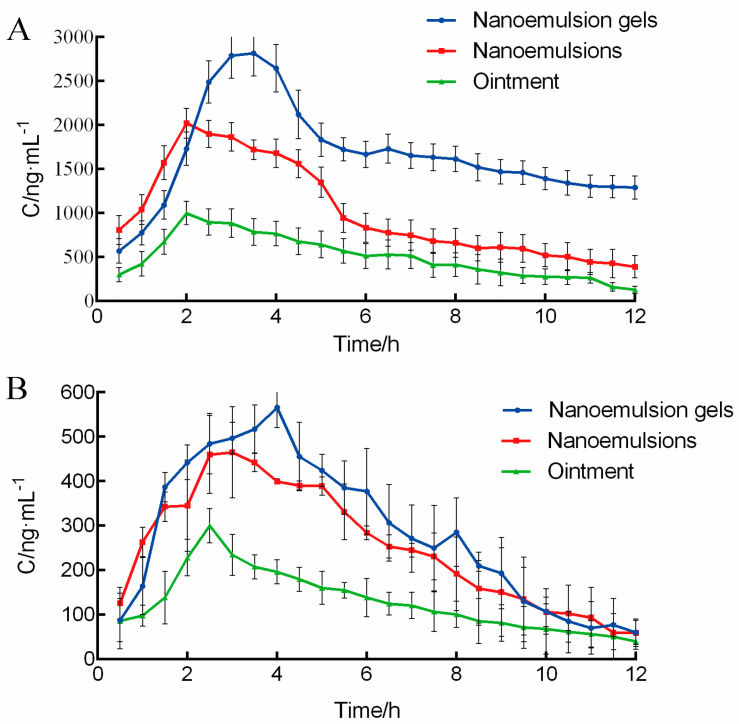
Triptolide concentration–time courses in skin (**A**) and blood (**B**).

A skin–blood synchronous microdialysis system was established. After the removal of abdominal hair from each group of rats, triptolide nanoemulsions, nanoemulsion−based gels and marketed triptolide ointments were administered to each group. Skin and blood MD samples were collected at each time point, and the triptolide concentrations in the dialysis solutions were detected with LC–MS. The actual concentrations of triptolide in the skin and blood were calculated using Formula (1), and the pharmacokinetic curve was drawn. The results are shown in Figure 2 and Table 3.

The results in Figure 2 indicate that the high concentration gradient appeared rapidly after the transdermal delivery of triptolide and reached the maximum concentration in 60 min. The transdermal rate was increased, and the time was decreased. The results above show that the concentrations of the triptolide nanoemulsion and triptolide nanoemulsion−based gels in the skin were significantly higher than those in the blood, and the concentrations of the marketed triptolide ointments in the skin and blood were both lower. This result indicates that the triptolide nanoemulsion and triptolide nanoemulsion−based gels both increased the triptolide concentrations in the skin and had better transdermal and local treatment effects [4,19]. Compared with the ointments, the nanoemulsion and nanoemulsion−based gels increased the triptolide concentration in subcutaneous tissue, and their transdermal effect was better; furthermore, the nanoemulsion−based gels had a sustained release effect. Compared with the ointments, the bioavailability of the nanoemulsion−based gels was comparable, and the blood concentration was more stable. Hence, for the treatment of inflammatory skin diseases, such as eczema, triptolide nanoemulsion−based gels can not only increase the triptolide concentration in the skin but also restrain the release of local inflammatory mediators, show a better local effect, maintain a higher bioavailability, and significantly decreased the systemic side effects of triptolide [20]. Compared with the nanoemulsion, the AUC of the nanoemulsion−based gels was significantly higher after transdermal delivery, and the triptolide concentration was more stable. The gels showed an obvious sustained release effect, and the bioavailability significantly increased. The reason might be the lower viscosity of the triptolide nanoemulsion−based gels, the poor adhesion on the surface of the skin, the better fluidity after administration, and the greater loss of dose. Blood concentration indicated that the bioavailability of the triptolide nanoemulsion−based gels was comparable to that of the triptolide ointments, but the blood concentration of the triptolide nanoemulsion−based gels was more stable, which significantly decreased the systemic side effects of triptolide.

### 2.2. Molecular Dynamics Simulation

**Figure 3:**
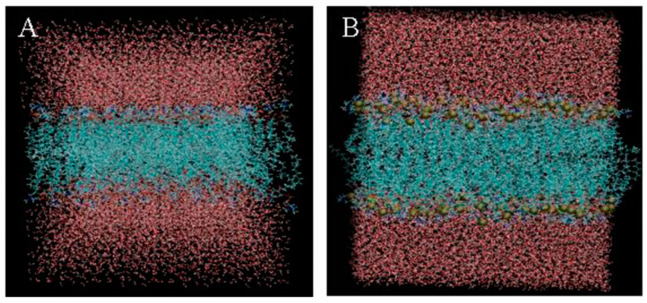
DPPC bilayer biofilm (**A**) and a new biofilm system of triptolide and DPPC (**B**).

The Charmmgui computing simulation platform was used to construct DPPC bilayer biofilms. The GROMACS force field parameters and the structure of the DPPC bilayer membrane after molecular dynamics equilibrium were obtained from the website of the Stockholm Laboratory. The completed bilayer biofilm is shown in Figure 3A, where a series of configuration files are generated. The simulation data of six energy balancing stages and one generation stage are shown in Table 4.

Triptolide is expressed in a spherical gold model. The head of the DPPC molecule in the cuticle phospholipid bilayer is represented by a spherical model, and the tail carbon chain is represented by a linear model, in which the carbon atom is green, the oxygen atom is red, the nitrogen atom is blue, and the phosphorus atom is gold. The water molecular layer is represented by the solvent surface model, which is located above and below the phospholipid bilayer.

For the initial simulated system, the steepest descent method was used for energy optimization to remove unreasonable atomic overlap. Then, a 100 ns molecular dynamics simulation was carried out. The specific parameters used in the simulation process are shown in Table 4.

**Figure 4:**
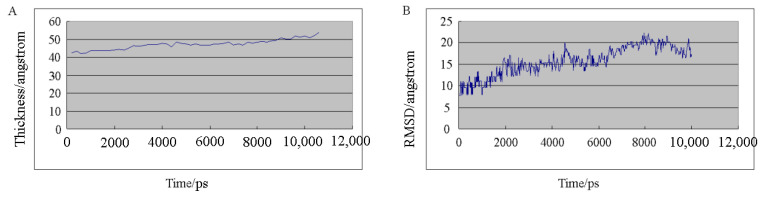
The simulation analysis results of DPPC biofilms: (**A**) Thickness; (**B**) *RMSD*.

The constructed DPPC bilayer biofilm was simulated and analyzed from the perspectives of temperature, kinetic energy, potential energy, pressure, and total energy (energy perspective), as well as the *RMSD* and film thickness. With the increase in the simulation time, the position of drug molecules relative to the membrane has a corresponding change, with a tendency to penetrate into the lipid membrane. The simulation analysis results of the DPPC biofilms are shown in Figure 4.

In the process of molecular dynamics simulation with the new system containing a DPPC biofilm and triptolide, the temperature, kinetic energy, potential energy, pressure, and total energy all changed within a certain range and tended to be stable. The *RMSD* value of the biofilm phospholipid molecules first was increased from small to large, finally stabilized and fluctuating at approximately 20. The thickness of the biofilm fluctuated gradually at approximately 50 angstroms during the simulation. The results show that the temperature, kinetic energy, potential energy, pressure, and total energy of the whole system were in equilibrium, the deviation degree of the atoms in the system was small, and the stability of the film was good.

**Figure 5:**
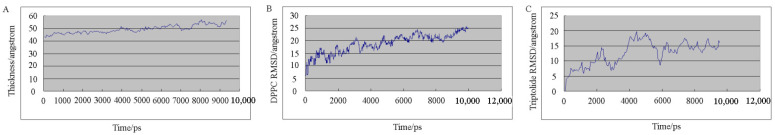
The simulation analysis results of the new system containing a DPPC biofilm and triptolide: (**A**) thickness; (**B**) *RMSD* of DPPC biofilm; (**C**) *RMSD* of triptolide.

The new architecture was built using Python programs, as shown in Figure 3B. The new system was simulated and analyzed in terms of temperature, kinetic energy, potential energy, pressure, total energy, *RMSD*, and film thickness. The simulation analysis results are shown in Figure 5**.** In the process of molecular dynamics simulation, the temperature, kinetic energy, potential energy, pressure, and total energy in a certain range of change tended to be stable and demonstrated the energy balance of the system as a whole. The molecular dynamics simulation results of the new system show that triptolide, as a new element added to the system, had little influence on the change in the energy of the whole system, and the *RMSD* of the biofilm in the new system showed no significant change, which indicates that the addition of triptolide to the system has no significant influence on the *RMSD* of the biofilm. The *RMSD* value of triptolide varied greatly and fluctuated violently, which indicates that triptolide molecules are constantly moving, with a large and irregular range of motion. The change in membrane thickness in the new system was similar to that in the previous biofilm simulation, indicating that the addition of triptolide had no effect on the change in biofilm thickness.

**Figure 6:**
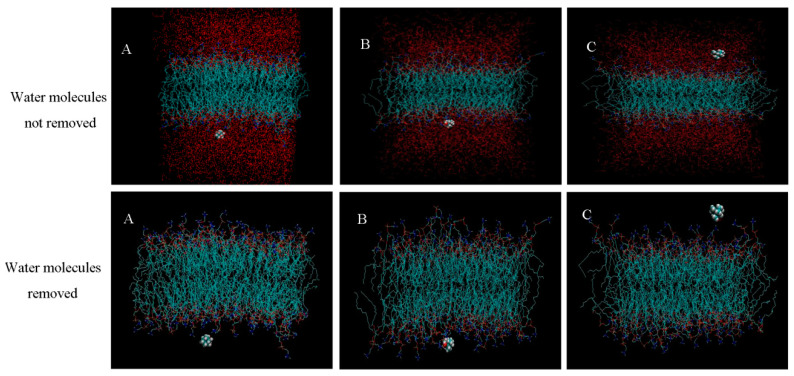
The simulated location of triptolide: (**A**) the initial location of the new system; (**B**) triptolide in contact with the phospholipid layer; (**C**) system diagram at the last moment.

Figure 6A shows the beginning of a whole system diagram of triptolide and biofilm, where it can be seen that triptolide is in the water molecules and moves towards the biofilm. The water molecules are red, and the phospholipid bilayer membranes surrounded by water molecules are blue. The relative position of the triptolide and bilayer membrane can be more clearly displayed after removing the water molecules. VMD shows that the position coordinates of triptolide are (35.758, 29.998, 23.602). Figure 6B shows that triptolide contacts and interacts with the biofilm during its movement in the water. It can be more clearly observed that triptolide contacts the biofilm in the molecular diagram of water removal, and the coordinates of triptolide at this time are (40.145, 25.609, 24.273). Figure 6C shows the last moment in the simulation process of the triptolide and biofilm. Within the simulation time, the triptolide molecules stay in the position shown in the figure. The system diagram of dehydrated molecules more intuitively shows the relative position of the triptolide and biofilm. At this time, the position coordinates of triptolide are (33.722, 21.450, 31.305).

### 2.3. Numerical Simulation

**Figure 7:**
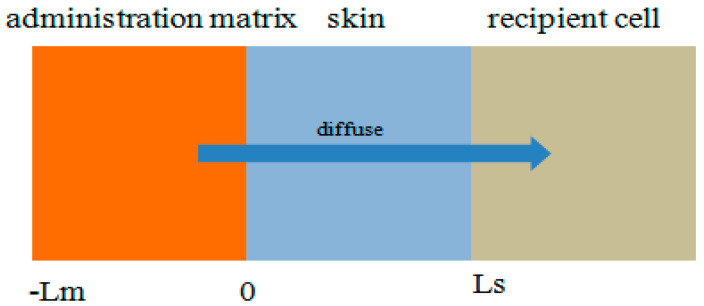
Multilayer structure model of the transdermal drug delivery system (L_m_ is carrier, and L_s_ is stratum corneum).

We established a multilayer structure model, and the whole model of the transdermal drug delivery system is shown in Figure 7.

The parameters of the drug diffusion coefficient in the different layers of the multilayer structure model are the key points for the numerical simulation. In this work, the diffusion coefficient in different skin layers was calculated using different methods, including MD simulation, ex vivo penetration tests, and in vivo skin microdialysis probe technology. The drug diffusion coefficient was calculated based on Fick’s law:

(3) $$D=-J/\left(\frac{\mathrm{dC}}{\mathrm{dx}}\right)$$

where C and X are known. Using the measurement data shown in Figure 1, the slope of the line is  J, and different formulation for the drug delivery system can be calculated in the matrix diffusion coefficient. Using the same method, microdialysis experiments were used to obtain the drug diffusion coefficient in the skin. In addition, the parameters $k_{m}$ = 0.5 and $k_{\mathrm{cl}}$ = 25 cm/h were obtained by reviewing the literature. The obtained model parameters are shown in Table 5.

**Figure 8:**
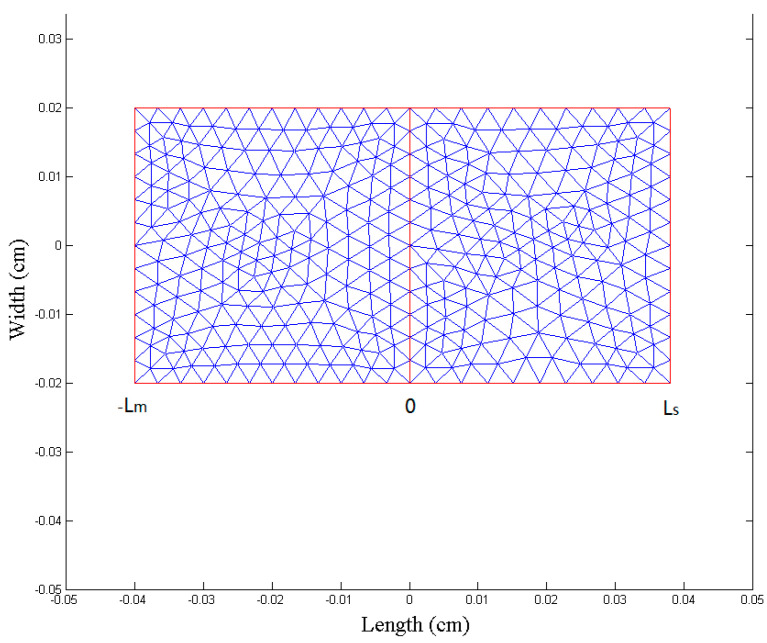
Finite elementary mesh of the finite element solutions.

FEA is a powerful grid based method for solving the partial differential equations of drug dynamic penetration in the skin. The node density varies in the domains and each point and node have a unique property. The mesh division, diffusion coefficient, and internal boundary conditions of the multilayer skin model are shown in Figure 8. The domain solution time was 10 h, and the time step length was 0.001 h = 0.6 min. The initial concentration of the drug in the model was C _mo_ = 0.2 g/cm^3^.

**Figure 9:**
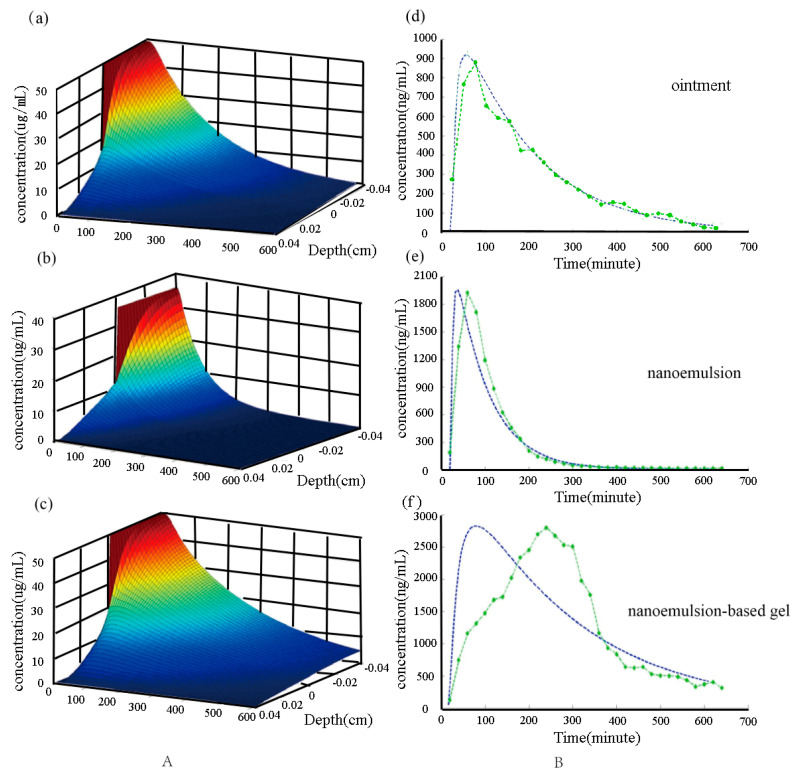
Two−layer model numerical simulation of the triptolide drug delivery system in a three−dimensional map: (**a**–**c**) ointment, nanoemulsion, and nanoemulsion−based gel, respectively. Comparison between experimental data and numerical results: (**d**–**f**) ointment, nanoemulsion, and nanoemulsion−based gel, respectively.

Figure 9, Group A shows the two−layer model numerical simulation of the triptolide drug delivery system in a three−dimensional map of the triptolide concentration variation with time and depth. Microdialysis can measure the drug concentration variation with time in the skin, and confocal microscopy was used to determine the variation in drug concentrations at a subcutaneous depth of 0.0336 cm. Group B shows the concentration curves of numerical simulation compared with the experimental data, where the blue dashed line is the numerical simulation results, and the green dashed line is the experimental data. For the ointment, we can see that the results of the numerical simulation are close to the practical measurements. For the nanoemulsion, we can see that only in the concentration increase phase does the numerical simulation result show some difference from the experimental results. For the nanoemulsion−based gel, we can see that the numerical simulation results are significantly different from the experimental results in both the concentration increase stage and the concentration decrease stage. The reasons for the different results in the numerical simulation and experimental data comparison in the three drug delivery system may be as follows: in the transdermal delivery model, the drug delivery matrix and skin are assumed to be homogeneous isotropic materials, but in fact, the skin is composed of the cuticle, epidermis, and dermis. It is clear that its diffusion coefficient is not a constant, and the drug concentration measurement by microdialysis is generally located in the dermis [21,22,23]. Therefore, the numerical simulation results and experimental results will exhibit certain deviations.

## 3. Materials and Methods

### 3.1. Materials

Capryol 90 was supplied by Gattefosse (Saint-Priest, France) as a gift. Tween 80 was supplied by Shanghai Lingfeng Chemical Reagent Co., Ltd. (Shanghai, China). OP−10 was obtained from Aladdin. Triptolide, with a purity of no less than 98.0%, was supplied by Shanghai Qingpu Chemical Co., Ltd. (Shanghai, China) (Lot 081201). The triptolide reference standard with a purity of no less than 98.0% was supplied by NICPBP (Lot 110708−200506). Methanol was obtained from Sigma−Aldrich (St. Louis, MO, USA) (HPLC grade). PBS (pH 7.4, containing Na_2_HPO_4_ 3.191 g/L, NaH_2_PO_4_ 0.775 g/L, and NaCl 5.58 g/L), a linear probe, concentric circle probe (in−house), an animal warm pad (in−house), and redistilled water were used. Other reagents were of AR grade.

### 3.2. Animals

The male Sprague Dawley rats of clean grade weighing 200 ± 20 g were approximately 1.5 months old. The animal study was approved by the Study Animal Centre of the Shanghai Ninth People Hospital, Shanghai Jiao Tong University School of Medicine. (SPF, SYXK 2016−0016).

### 3.3. Experimental Method

#### 3.3.1. LC–MS Analysis [4,5]

Chromatographic conditions: ZORBAX Extend−C18 (2.1 mm × 100 mm, 3.5 um) chromatographic column; mobile phase: 0.1% (*v*/*v*) formic acid aqueous solution/methanol = 40:60; flow rate: 0.3 mL/min; column temperature: 40 °C; injection volume: 3 μL.

Mass spectrum conditions: ion source: ESI source; scanning mode: MRM, cation detection; ion for quantitative analysis: *m*/*z* = 361.3/105.2; dry gas flow rate: 8 L/min; dry gas temperature: 350 °C; capillary voltage: 4000 V; atomizer pressure: 40 psi; cracker voltage: 105 eV; collision energy: 33.

A triptolide stock solution at a concentration of 1 μg/mL was used to prepare the standard solutions at concentrations of 10 ng/mL, 100 ng/mL, 200 ng/mL, 300 ng/mL, 400 ng/mL, and 500 ng/mL. The above solutions at different concentration levels were injected and detected to record the peak area. The standard curve was obtained by linear regression with the triptolide peak area (Y) and concentration (X). The standard curve of triptolide was Y = 3.3827X − 12.053, r = 0.9999, and the linear range was 10–500 ng/mL.

Samples of the triptolide solution were measured at 0, 2, 4, 6, and 8 h, and the RSD value was 0.29%, indicating that the samples were stable within 8 h. The samples with concentrations of 10 ng/mL, 100 ng/mL, and 500 ng/mL were measured at 0, 4, 8, 12, and 24 h and 1, 2, 3, and 4 days. The intra−day RSDs were 4.70%, 1.79%, and 0.75%, respectively, and the intra−day precision RSDs were 5.55%, 2.46%, and 0.45%, respectively. The results show that the method has good accuracy. Then, triptolide at low, medium, and high concentrations was added to the sample (10 ng/mL, 100 ng/mL, and 500 ng/mL) for the determination and calculation of the recovery rates; the recoveries were 98.53%, 99.76%, and 100.05%, respectively, and the RSDs were 2.88%, 0.24%, and 0.29%, respectively. The results showed that the method has a good recovery rate (for more details on the chromatographic separation and method please refer to the Supplementary Materials).

#### 3.3.2. Preparation of Nanoemulsion and Nanoemulsion−Based Gels

According to the formula of the triptolide nanoemulsion [4], triptolide was dissolved in Capryol 90, OP−10 was added as a surfactant, and Tween 80 was added as a co−surfactant and fixed to obtain the internal phase-containing drug. Water was slowly added to the internal phase at room temperature with stirring at a constant speed of 300 r/min until clear. Carpol 940 was taken and added to glycerol to grind and wet, and then the triptolide nanoemulsion prepared as described above was slowly added and left to swell. Triptolide nanoemulsion−based gels were prepared.

#### 3.3.3. Ex Vivo Percutaneous Penetration

The abdomens of the SD rats (Male, 200 ± 20 g) were depilated one night before the experiment, and the rats were euthanized the next day. The abdominal skin of the SD rats was scraped clean with a scraper for standby. A Franz diffusion cell was used, and the receiving solution was PBS (pH = 7.4, containing 20% absolute ethanol). Then, 0.5 g of TPL nanoemulsions and nanoemulsion−based gels or ointment was placed into the supply cell of the Franz diffusion cell, and the transdermal experiment was conducted at the condition of 37 ± 1 °C, 300 r/min; the samples were taken at the specified 1 h time point. At 2, 4, 6, 8, 10, and 12 h, 1 mL of receiving solution was removed (adding 1 mL PBS at the same time), the sample was centrifuged at 10,000 r/min for 5 min, and the supernatant of the sample was taken after centrifugation for detection. Then, the cumulative percutaneous permeability Qn area was calculated as follows:

Qn = (CnVn + ∑CiVi)/A(4)

where Cn is the drug concentration at the n point, Ci is the drug concentration at the i point, Vn is the volume of the receiving tank (5 mL), Vi is the sampling volume (1 mL), and A is the diffusion area of the diffusion tank (0.785 cm^2^). The slope of the regression line dQn/dt obtained by the linear regression is the steady−state penetration rate *J_s_* (ng·cm^−2^·h^−1^).

#### 3.3.4. In Vivo Microdialysis Studies

The ex vivo recovery of the skin linear probe and blood vessel concentric circle probe affected by the infusion speed was investigated with the gain method and loss method [4]. The infusion speed of the infusion fluid was chosen as 5 μL/min.

Gain method: The “efficient dialysis window” of the linear MD probe (membrane length of probe 20 mm. intercepted relative MW 5000 Dalton) or concentric circle MD probe (membrane length of probe 10 mm. intercepted relative MW 5000 Dalton) was immersed in PBS containing triptolide with three different concentrations (C in: 52.5, 210, or 1050 ng/mL). The magnetic stirrer was operated at 200 rpm, and the temperature of the water bath was 37 ± 0.5 °C. The infusion fluid was PBS, and the infusion speed was 5 μL/min, each group was equilibrated for 1 h, and the dialysis solution was collected every 30 min. The triptolide concentrations (C out) in the dialysis solutions were detected with LC–MS. However, the loss of PBS and infusion fluid was reversed. The gain method recovery followed formula (5), and the loss method recovery followed formula (6):

(5) $$Recovery\;(\%)=\;\frac{C\;\mathrm{out}}{C\;\mathrm{in}}\;\times \;100\%$$

(6) $$Recovery(\%)=\;\frac{C\;\mathrm{in}-C\;\mathrm{out}}{C\;\mathrm{in}\;}\;\times \;100\%$$

Next, we measured the in vivo recovery rate of the microdialysis probe. First, the abdomens of the SD rats were depilated with depilatory ointment. After anesthetizing the rats, they were fixed on a heat preservation pad (to maintain the temperature of the rats at 37.5 °C), and then the linear probe was guided into the subcutaneous tissue of the rats’ abdomen with a puncture needle and drawn. An opening was created in the rats’ neck to bluntly separate the jugular vein and then a surgical line was used to was used ligate the distal end of the jugular vein. After ligation, an artery clamp to was used clamp the end of the jugular vein close to the heart, and a knife was used to open the jugular vein; afterwards, the artery clamp was released quickly, the concentric probe was inserted into the heart along the jugular vein, and then the concentric probe was ligated to the jugular vein to fix the probe. Finally, the wound on the neck was sutured.

After the operation, the “dialysis membrane” of the skin linear probe was placed into PBS solution (without drugs), and the perfusion rate was 5 μL/min. PBS (containing 52.5, 210, and 1050 ng/mL triptolide) was used to infuse the probe, and the in vivo recovery rate of the skin linear probe was calculated according to Formula (5). In the blood vessel concentric probe, the solution of the infusing probe was PBS (containing 52.5, 210, and 1050 ng/mL triptolide) and the recovery rate of triptolide in the concentric vascular probe was calculated according to Formula (6). Formula (7) was used to correct the in vivo recovery rate obtained, and Formula (8) was used to calculate the concentration of triptolide in vivo in the pharmacokinetic process.

(7) $$\left(R/D\right)\mathrm{in}\;\mathrm{vivo}=\left(R/D\right)\mathrm{ex}\;\mathrm{vivo}$$

C in vivo = C ex vivo /R in vivo(8)

where R is the recovery rate obtained using the gain method and D is the recovery rate using the loss method.

Finally, we conducted a pharmacokinetic experiment on the SD rats [4,5]. The surgical procedure was the same as the procedure for the recovery rate in vivo. After the operation, we used black PBS as the infusion solution with an infusion the speed of 5 μL/min and equilibrium after 1 h. The rats of each group were administered 1% triptolide nanoemulsion, triptolide nanoemulsion−based gels, and marketed triptolide ointments. The dose was 1 g, and the area was 3 × 4 cm^2^. The area was covered with a polyethylene membrane. The dialysis solution was collected every 30 min, and the experiment lasted for 12 h. The triptolide concentrations in the dialysis solutions were detected with LC–MS.

#### 3.3.5. Pharmacokinetic Analysis

The data are shown as x ± s. The data were analyzed with Kinetica 5.0 software to obtain the pharmacokinetic parameters.

### 3.4. Molecular Dynamics Simulation

#### 3.4.1. Construction of DPPC Bilayer Biofilms

Using the CHARMM−GUI platform to build a DPPC bilayer organism, the following steps were followed to build a membrane: read the coordinates, determine the orientation, determine the size of the system, establish the composition, assemble thecomposition, and balance the system of the lipid bilayer [24,25,26]. During the modeling process, the selection of relevant parameters in the system was as follows: the upper and lower layers of the DPPC bilayer were 100 DPPC molecules, and each DPPC lipid molecule was attached to 50 water molecules. The sizes of the upper and lower layers of DPPC lipids were 6300 g. The size ratio of the *X* axis to *Y* axis was selected as 1:1. M NaCl (physiological ion concentration) was selected to ensure the electrical neutrality of the system. Finally, a series of input files were generated, which could be divided into job descriptions, adjustable parameters, simulation parameters, extra parameters, execution scripts, etc [27].

We analyzed the DPPC bilayer biofilms from three perspectives with the help of Xshell software. The energy angle includes multiple temperatures, kinetic energy, potential energy, pressure, and total energy. The *RMSD* value is used to measure the degree to which an atom deviates from its alignment position [28]. The formula for solving the *RMSD* value of atomic α in N_t_ in a certain period of time is shown in Formula (9):

(9) $$RMSD_{\alpha }\left(t_{j}\right)=\sqrt{\frac{\sum_{\alpha =1}^{N_{\alpha }}{\left(\vec{r_{\alpha }}\left(t_{j}\right)-\langle \vec{r_{\alpha }}\rangle \right)}^{2}}{N_{\alpha }}}$$

where Nα is the number of atoms involved in the comparison, the position of atom α at the current moment, and the average position of atom α during the whole time period, which is defined as:

(10) $$\langle \vec{r_{\alpha }}\rangle =\frac{1}{N_{t}}\sum_{j=1}^{N_{t}}\vec{r_{\alpha }}\left(t_{j}\right)$$

N_t_ is a time range in steps.

In this simulation, the *RMSD* value reflects the degree of deviation of each atom of the biofilm from the average position, which is the size of the motion amplitude of each atom. The *RMSD* value is increased with the spatial range of the atom motion [29,30].

#### 3.4.2. Construction of a New Biofilm System of Triptolide and DPPC

We consulted the works [31,32,33] and simulated a new bilayer system of triptolide and DPPC phospholipid. The simulation method is as follows: first, the central coordinates of the upper layer of the bilayer membrane need to be determined. The molecular P site reference at the outermost side of the bilayer biofilm is selected, then a P atom at the outermost side of the *X* axis and *Y* axis is chosen, and, finally, the highest water molecule in the *Z* axis direction is selected. Half of the *X* coordinate value of atom P in the first row added to the second row is recorded as A. Half of the *Y* coordinate value of atom P in the first row i plus the value in the third row is recorded as B. Half of the *Z* coordinate value in the first row of atom P added to the fourth row of water molecules is recorded as C. The coordinates (A, B, C) are the central coordinates of the upper layer of the bilayer film.

After the triptolide is moved onto the DPPC bilayer biofilm, it is necessary to remove the water molecules in the part where the triptolide contacts the DPPC bilayer biofilm to obtain a stable new system of triptolide and DPPC bilayer biofilm, which is ready for the molecular dynamics simulation of the new system. To determine the size of triptolide, the *X*, *Y*, and *Z* coordinates of triptolide are moved behind the central coordinate of the upper layer of the DPPC bilayer biofilm to find their maximum and minimum values, and then 2 is added to the maximum value and 2 is subtracted from the minimum value, so as to ensure that there are no water molecules within the range of triptolide molecules. Secondly, the *X*, *Y*, and *Z* coordinates of all water molecules in the DPPC bilayer biofilm are compared with the previously determined range of each coordinate of triptolide. When the *X*, *Y*, and *Z* coordinates of the water molecule meet the coordinate range at same time, this water molecule is deleted, so that there is no water molecule in contact with the triptolide and DPPC bilayer biofilm. Following this, anew system of stable triptolide and DPPC bilayer biofilm is obtained.

The simulation analysis of the new system was still carried out from three perspectives, namely, angle, *RMSD*, and film thickness. During the simulation of the triptolide molecules and DPPC biofilms, the triptolide molecules continued to move and change their positions, as well as contacting the phospholipid layer of the DPPC biofilms, which allowed us to observe the movement trajectory of the two molecules.

### 3.5. Numerical Simulation

#### 3.5.1. Establishment of Multilayer Structure Model

The transdermal drug delivery system is composed of three parts, namely, the drug matrix, the skin, and receptor cells. Because the drug matrix and skin physical properties, especially the drug diffusion coefficient and initial drug concentration, have significant differences; the whole drug delivery system is divided into three layers: the first layer is the drug matrix, the second layer is the skin, and the third layer is the dermis−linked subcutaneous tissue and capillaries.

Researchers in article [34,35,36,37] used the diffusion equation to establish the process of drug permeation through the skin surface into the skin. The drug concentration in the matrix is satisfied with the diffusion equation.

(11) $$\frac{\partial^{2}C_{m}}{\partial t}=D_{m}\frac{\partial^{2}C_{m}}{\partial t}-L_{m}\leq x\leq 0,\;t>0$$

where $D_{m}$ indicates the diffusion coefficient of the drug in the matrix. At the same time, if the thickness of the skin is $L_{s}$, the concentration of the drug $C_{s}$ can also satisfy the diffusion equation.

(12) $$\frac{\partial C_{s}}{\partial t}=D_{s}\frac{\partial^{2}C_{s}}{\partial x^{2}}\;0\leq x\leq L_{s},\;t>0$$

where $D_{s}$ indicates the diffusion coefficient of the drug in the skin.

For the surface of the substrate, the boundary conditions are satisfied because there is no inflow or outflow of drugs.

(13) $$\mathrm{When}\;x=-L_{m}\frac{\partial C_{m}\left(-L_{m},t\right)}{\partial x}=0\;0\leq t\leq T$$

At the interface of the drug administration and skin, the drug’s inflow or outflow satisfied the conditions of convergence.

(14) $$\mathrm{When}\;x=0-D_{m}\frac{\partial C_{m}\left(0,t\right)}{\partial x}=-D_{s}\frac{\partial C_{s}\left(0,t\right)}{\partial x}\;0\leq t\leq T$$

(15) $$\mathrm{When}\;x=0k_{m}C_{m}\left(0,t\right)=C_{s}\left(0,t\right)\;0\leq t\leq T$$

where $k_{m}$ is the isolation coefficient of the interface between the drug delivery matrix and the skin. The drug eventually enters the skin and is absorbed by the capillary. We regard capillary absorption as a boundary condition.

(16) $$\mathrm{When}\;x=L_{s}-D_{m}\frac{\partial C_{s}\left(L_{s},t\right)}{\partial x}=k_{\mathrm{cl}}C_{s}\left(L_{s},t\right)\;0\leq t\leq T$$

where $k_{\mathrm{cl}\;}$ is the clearance rate of the basic capillary concentration. In addition, the initial condition of the model is that the uniform distribution of drug concentrations is $C_{m0}\;$ in the drug delivery matrix, while the initial drug concentration in the skin is $C_{s0}$:

(17) $$\mathrm{When}\;t=0{\;C}_{m}\left(x,0\right)=C_{m0}-L_{m}\leq X\leq 0$$

(18) $${\;C}_{m}\left(0,t\right)=C_{s0}\left(0,t\right)\;0\leq X\leq L_{S}$$

#### 3.5.2. Selection of Model Parameters

Obtaining the diffusion coefficient in the multilayer structure model is the key to the numerical simulation. Using ex vivo drug diffusion experiments and in vivo microdialysis experiments, data on the interface can be obtained by fitting the experimental data to obtain the diffusion coefficient of the drug in the matrix and the skin. The specific method is as follows:

(19) $$J=-D\frac{\mathrm{dC}}{\mathrm{dx}}$$

where J is the drug through the interface (unit is μg/cm^2^/h); C is the drug concentration in the matrix (unit is μg/cm^3^); X is the matrix of the thickness (unit is cm); and D is the drug diffusion coefficient in the matrix.

#### 3.5.3. Numerical Methods

We used the partial differential equation in MATLAB 7.5 to solve the diffusion equation of the PDETool toolbox. The solution region is defined as a rectangular region: width W = 0.04 cm and length L = L_s_ + L_m_ = 0.08 cm, where L_s_ = 0.04 cm and L_m_ = 0.04 cm.

## 4. Discussion

The pharmacokinetics of TPL was studied using in vivo skin vessels. Nanoemulsions and nanoemulsion gels display a trend at the application site, which shows that the transdermal drug absorption of the two nano−formulations is similar. Nanoemulsion and nanoemulsion gels cause an enclosure effect and the hydration of the keratin layer, enhancing the transdermal permeation of the drug [38]. By forming a lipid film on the stratum corneum, the drug penetrates the skin, and the absorption of the drug can be enhanced [39]. In addition, drug molecules must first be released from nanoparticles to be absorbed, which may cause fluctuations in the level of drug in the skin [40].

From the microdialysis in/ex vivo recovery experiment and the correction of the recovery rate, it can be seen that the recovery of the probe measured using the gain method and loss method was basically consistent. The recoveries of the skin linear probe and blood vessel concentric probe measured using the gain method and loss method were basically the same under different drug concentrations, which shows that the recoveries of the two probes were almost independent of drug concentrations. The corrected in vivo drug recovery can be used to calculate the pharmacokinetics process of the drug in vivo. Due to the complexity of the plasma composition, there are many substances in the serum (plasma), so it is difficult to isolate and identify the drug in plasma. On the other hand, the skin possesses a rich enzyme system, and it metabolizes during the transdermal delivery process; thus, this component is more complex. Therefore, the drug concentration is extremely low and difficult to detect using traditional methods. Skin–blood dual−site simultaneous microdialysis technology provides a new research method for the pharmacokinetics of triptolide in vivo. Compared with traditional blood samples, the tissue fluids obtained from the simultaneous microdialysis sampling of the skin and blood sites contain “signal molecules” that have been screened by dialysis membrane filtration, which can more realistically reflect the drug disposal situation with almost no damage to the skin [41,42]. The advantages of this technology in vivo, such as in situ sampling and online, the real−time detection make it particularly suitable for transdermal drug delivery studies.

At present, the research on the mechanism of nanoparticles in promoting drug percutaneous absorption mainly uses ex vivo percutaneous diffusion cells based on animal skin. Visually, this reflects the interaction between the drug carrier and the skin but cannot directly show the interaction between the lipid nanocarrier and cuticle [43]. It is difficult to deeply explain the mechanism of transdermal drug delivery. However, the mechanism of transdermal drug delivery is very important for drug design and development. Therefore, a model of nanocarriers and the lipid membrane in the stratum corneum was constructed by using molecular dynamics. It describes the dynamic process of lipid nanocarrier visually and dynamically and can obtain the important information that cannot be measured in the experiment. Additionally, the experimental data can be used to check and correct the theoretical simulation. As a result, the combination will deeply help people to understand the relationship between the microstructure and macro performance of the research [44].

In the past, mathematical modeling was carried out in a variety of ways. Many studies used pharmacokinetic models based on one or more (thorough agitation) compartments that describe skin layers and carriers. The drug concentration in each layer was molded from a conventional differential equation that does not provide information about the depth concentration distribution in the skin. Our mathematical method is based on a diffusion model, which consists of a drug delivery partial differential equation that describes the space and time according to Fick’s diffusion law. Since they are physical models, all parameters (diffusion coefficients, allocation factors) have physical and systematic independent meanings, allowing independent parameter identification in the system. This study proposes a two−dimensional diffusion model and compares it with experimental results. On the basis of the multicomponent transport diffusion model that was developed [45,46], we studied the correlation between the penetrations of different carriers through the skin to describe the effects of penetrating modifications. Due to the properties of the diffusion equation solution, both the skin and administration matrix are assumed to be homogeneous isotropic materials, in fact, the skin is composed of the stratum corneum, the epidermal layer and the dermis layer. Therefore, its diffusion coefficient is apparent, and the concentration of the drug measured by microdialysis technology is generally located in the dermis layer, which is not realistic; thus, there will be some deviation between the numerical simulation results and the experimental results.

This project is based on the combination of molecular dynamics simulation and experimental research, with triptolide as a model drug to build the model of a nanocarrier for transdermal drug delivery and the lipid membrane in the stratum corneum. The interaction between the lipid nanocarrier and stratum corneum is studied from the microscopic and mesoscopic scales using molecular dynamics simulation methods, and the dynamic process of the lipid nanocarrier penetrating the skin is described intuitively and dynamically. We compare three formulations of triptolide to analyze the results of the experimental research and computer simulation to clarify the mechanism of the nanocarrier. The experimental research combined with the mathematical modeling provides a good research method for triptolide transdermal delivery systems.

## 5. Conclusions

This study investigated the pharmacokinetic course in the skin and blood after the transdermal delivery of triptolide using the MD technique, discussed the effect of probe recovery on the triptolide concentration at the aphotic infusion speed, and confirmed that triptolide has the same recovery and delivery for the skin linear probe and concentric circle probe. Therefore, the triptolide concentrations in the skin and blood can be detected with MD combined with LC–MS. We initially established the pharmacokinetic research on triptolide transdermal delivery based on the skin–blood synchronous microdialysis technique, obtained the triptolide concentration time curve in the skin and blood after transdermal delivery in rats, and provided a new idea and method for pharmacokinetic research on transdermal delivery. Triptolide nanoemulsion−based gels might be expected to be a new formulation for transdermal delivery.

This study shows that the three−layer model can be used for transdermal drug delivery system drug diffusion research; numerical simulation with parameter modification is convenient, accurate, and inexpensive. Therefore, it is conducive to transdermal drug delivery system design and optimization of the dosage form. Based on the drug concentration of the in vivo microdialysis measurement technology, the diffusion coefficient of the drug in the skin can be more accurately measured, and the numerical results can be verified. Therefore, the microdialysis technique combined with mathematical modeling provides a very good platform for the further study of transdermal delivery systems.

## Figures and Tables

**Table 1:** Results of triptolide concentrations on the ex vivo recovery determined by the gain method and loss method from the linear microdialysis probe and concentric cannula microdialysis probe (*n* = 5).

Concentration (ng/mL)	Linear Recovery (%)		Concentric Cannula Recovery (%)	
	Gain Method	Loss Method	Gain Method	Loss Method
52.5 (low)	61.4 ± 2.4	59.2 ± 3.1	48.4 ± 6.3	48.9 ± 3.8
210 (middle)	61.4 ± 1.9	63.8 ± 1.8	53.7 ± 1.8	59.1 ± 4.5
1050 (high)	62.1 ± 2.2	64.4 ± 3.8	49.1 ± 0.5	54.1 ± 1.5

**Table 2:** Results of triptolide concentrations on in vivo recovery determined by the loss method from the linear microdialysis probe and concentric cannula microdialysis probe (*n* = 5).

Concentration (ng/mL)	Linear Recovery (%)	Concentric Cannula Recovery (%)
21	/	46.4 ± 9.9
52.5	64.8 ± 5.5	54.1 ± 4.7
210	70.9 ± 3.5	54.4 ± 3.7
1050	72.4 ± 2.2	/

**Table 3:** Pharmacokinetic parameters in skin and blood after three triptolide delivery system administrations (mean ± SD).

Parameters	Nanoemulsion		Nanoemulsion Gel		Ointment	
	Skin	Blood	Skin	Blood	Skin	Blood
C_max_ (ng/mL)	2020.21 ± 170.27	464.98 ± 102.54	2645.43 ± 269.45	565.92 ± 45.66	998.72 ± 133.70	300.01 ± 38.82
T_max_ (min)	120.0	180.0	210.0	240.0	120.0	150.0
T_1/2_ (min)	363.8 ± 20.5	223.5 ± 60.1	185.6 ± 29.1	234.8 ± 19.2	189.2 ± 35.1	234.6 ± 54.7
AUC_0–660_ (ng·min/mL)	13,412,917 ± 109,463.9	116,743.4 ± 98,612.4	17,674,876.1 ± 132,437.9	202,961.8 ± 18,672.8	8,764,515.3 ± 23,977.3	97,210.4 ± 20,966.4
AUC_0–∞_ (ng·min/mL)	18,932,186.3 ± 119,216.3	134,865.2 ± 89,192.0	21,126,376.4 ± 152,863.4	247,136.4 ± 17,604.1	9,007,619.4 ± 22,409.1	108,876.2 ± 36,214.6

**Table 4:** Simulation parameters in molecular dynamics simulation.

Name	Parameter
System	NTP (constant temperature and pressure, and the number of particles in the system remains unchanged)
Periodic boundary condition box	6 nm × 6 nm × 6 nm
Force field	GROMACA force field
Temperature	323 K, Nose Hoover temperature coupling, coupling constant 0.1 ps
Pressure	1 bar, Parrinello Rahman pressure coupling, coupling constant 1 ps
Time interval of each step	2 fs
Covalent bond constraint Electrostatic interaction Van der Waals interaction	LINCS algorithm Particle mesh Ewald scheme with 10 Å cut−off Lennard−Jones interactions with 10 Å cut−off

**Table 5:** Parameters of the numerical simulation model.

Dosage Form	Dm (× 10^−6^ cm^2^/h)	Ds (× 10^−6^ cm^2^/h)
Ointment	9.21	20.25
Nanoemulsion	15.72	46.15
Nanoemulsion−based gel	7.42	10.60

## Data Availability

The data that support the finding of this study are available from the corresponding author upon reasonable request.

## Supplementary Materials

The following supporting information can be downloaded at: https://www.mdpi.com/article/10.3390/molecules28020553/s1, Figure S1: Full scan mass spectrum of TPL; Figure S2: Fragment ion scan mass spectrum of TPL; Figure S3: Choice of flow-rate of drying gas; Figure S4: Choice of nebulizer pressure; Figure S5: Choice of fragmentor voltage; Figure S6: Choice of collision energy; Figure S7: Mass spectrum of lactone armor (a), TPL nanoemulsions (b), TPL nanoemulsion gels (c) and TPL gels (d); Figure S8: LC/MS standard curve of TPL; Table S1: Results of stability test (*n* = 6); Table S2: Results of intraday precision test (*n* = 6); Table S3: Results of Daytime precision test (*n* = 6); Table S4: Results of recovery test (*n* = 6).
