# Supplementary material for: Pharmacokinetic Study of Triptolide Nanocarrier in Transdermal Drug Delivery System—Combination of Experiment and Mathematical Modeling
Source: Molecules. 2023 Jan 5;28(2):553. doi: 10.3390/molecules28020553 (PMC9866283; doi:10.3390/molecules28020553)
Supplement: Supplementary file 1 [file molecules-28-00553-s001.zip › molecules-2099763-supplementary.pdf]

LC/MS Analysis

Chromatographic condition

ZORBAX Extend-C18 column (2.1 mm × 100 mm, 3.5 um) chromatographic column; Mobile phase: 0.1% (v/v) formic acid aqueous solution: methanol = 40: 60; Flow rate: 0.3 ML/min; Column temperature: 40 ℃; Injection volume: 3 L.

Mass spectrum conditions

Ion source: ESI source; Scanning mode: MRM, cation mode detection; Ion for quantitative analysis: *m*/*z* = 361.3/105.2; Dry gas flow rate: 8 L/min; Dry gas temperature: 350 ℃; Capillary voltage: 4000 V; Atomizer pressure: 40 psi; Cracker voltage: 105 eV; Collision energy: 33.

(1) Selection of excimer ions

In the full scan mass spectrum of triptolide cation mode, its main composition is [M+H] peak, and its excimer ion peak is *m*/*z* = 361.3.

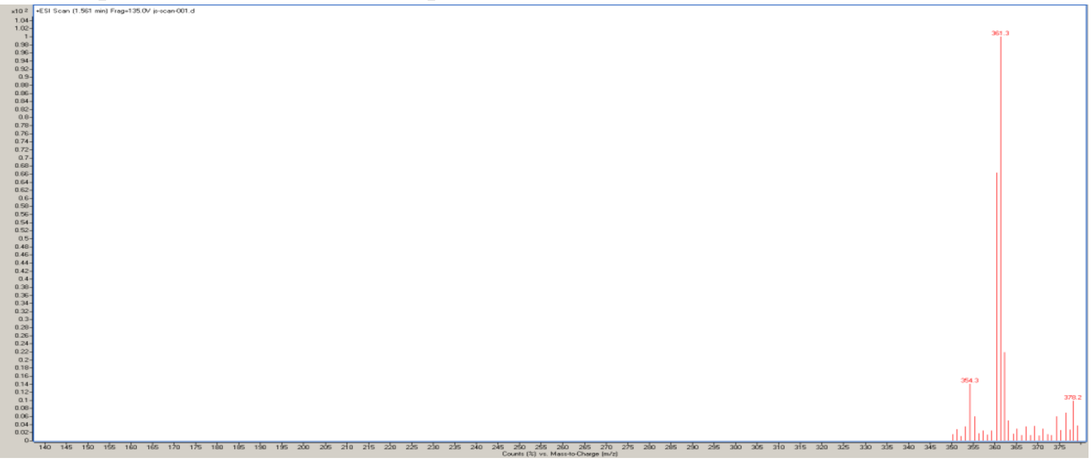

Figure S1. Full scan mass spectrum of TPL

(2) Selection of fragment ions

The fragment ions were further scanned for quantitative analysis and the fragment ions with *m*/*z* = 105.2 had the highest and stable response value. Therefore, fragment ions with *m*/*z* = 105.2 were selected as monitoring ions.

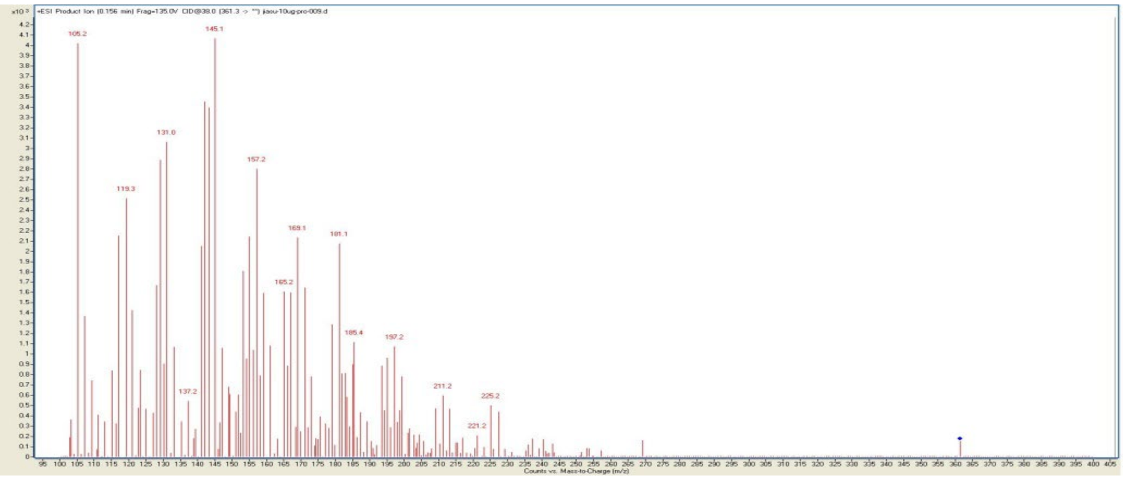

Figure S2. Fragment ion scan mass spectrum of TPL

(3) Selection of drying gas flow rate

The effects of different drying gas flow rates of 8 L/min, 10 L/min and 12 L/min on the mass spectrum response of triptolide were investigated. The results showed that the response value was the highest when the drying gas flow rate was 8 L/min, finally 8L/min was selected as the drying gas flow rate.

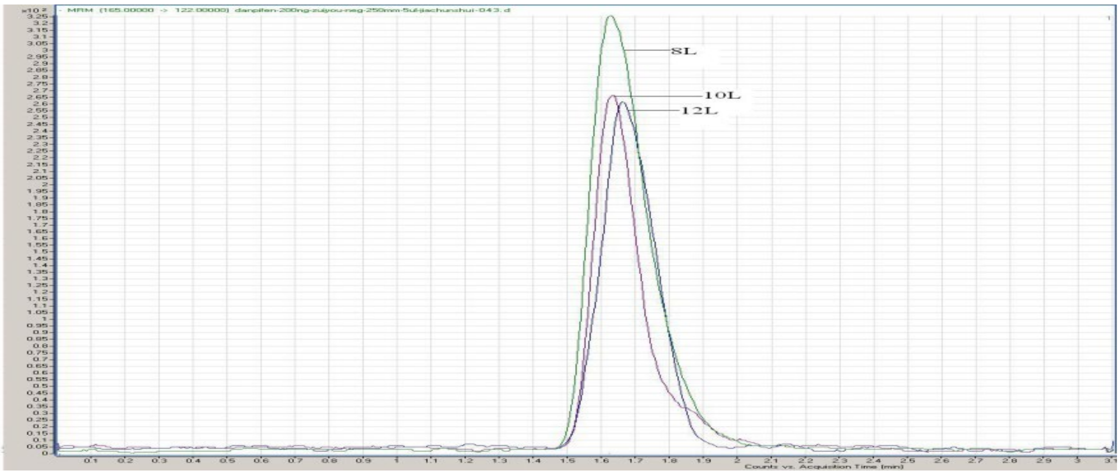

Figure S3. Choice of flow-rate of drying gas

(4) Selection of atomization pressure

The influence of atomizer pressure of 30 psi, 40 psi and 45 psi on the mass spectrum response of triptolide was investigated. The results showed that the mass spectrum response was the highest when the atomizer pressure was 40 psi, so the atomization pressure is chosen 40 psi.

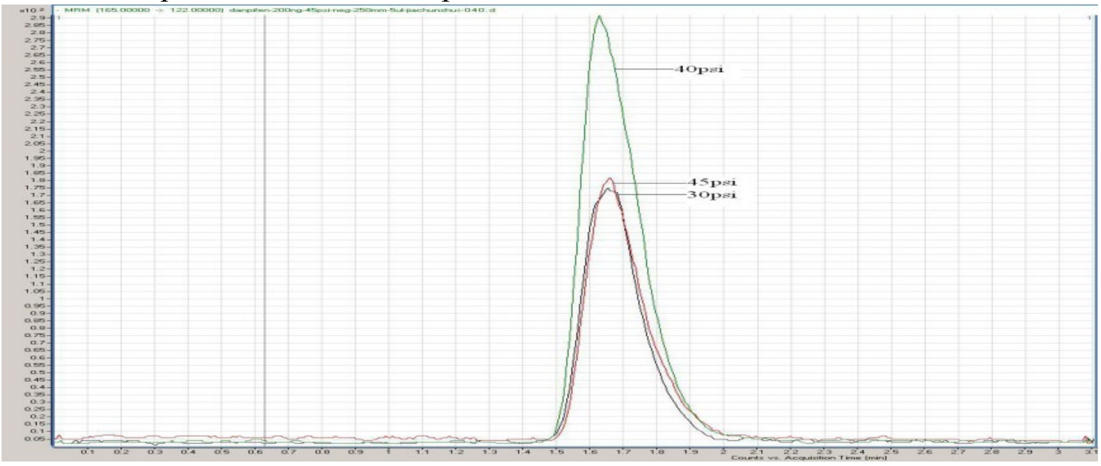

Figure S4. Choice of nebulizer pressure

(5) Selection of cracking voltage

The effects of cleavage voltages 103, 105, 110 and 120eV on the diathesis spectrum response of tripterygium wilfordii were investigated. The results showed that the highest mass spectrum response value was obtained when the cleavage voltage was 105 ev.

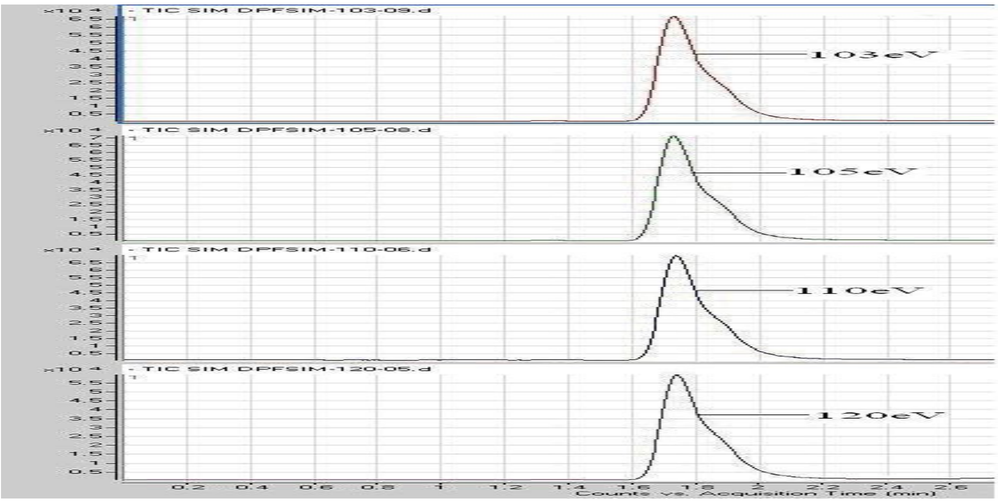

Figure S5. Choice of fragmentor voltage

(6) Choice of collision energy

The effect of collision energy 29, 30, 31, 32, 33, 34 on the mass spectrum response of triptolide was investigated. The results showed that the mass spectrum response value was the highest when the collision energy was 33.

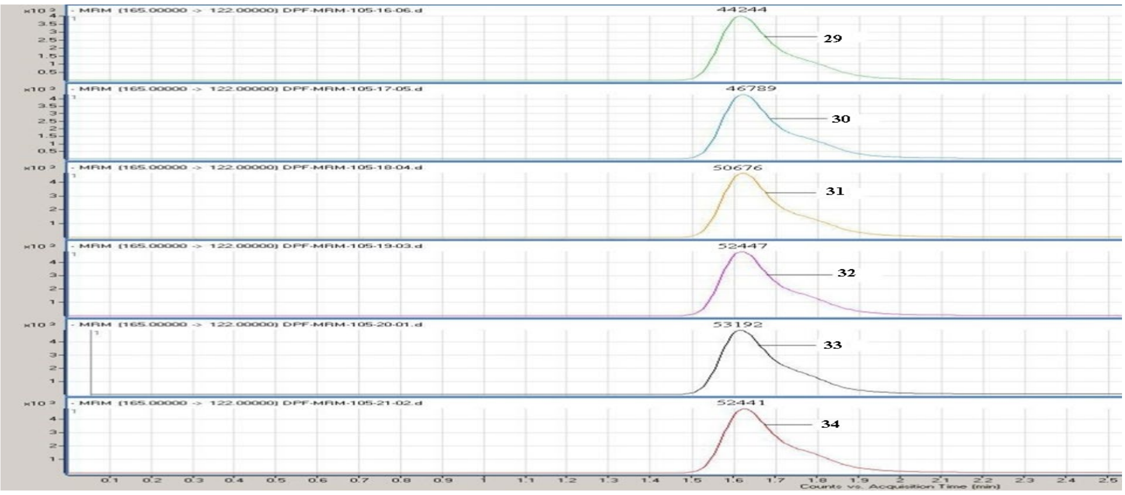

Figure S6. Choice of collision energy

Investigation of specificity

Preparation of reference solution: 5 mg of triptolide reference solution was accurately weighed and placed in a 100 mL measuring bottle, then dissolved in methanol and diluted to the scale by ultrasound. Then, 2 mL of the solution was carefully measured and placed in a 100 mL measuring bottle to obtain a 1 μg/mL reference solution, which was sealed with a sealing film and stored in a refrigerator at 4 ℃.

Preparation of sample solution: The prepared triptolide nanoemulsion, triptolide nanoemulsion gel, and triptolide ordinary gel 1g were respectively placed in a 10mL volumetry bottle, then demulsified with 5mL methanol and sonized for 30min. The samples were further diluted with methanol to the scale, and then placed in a low-temperature high-speed centrifuge at 10000 r/min for 5 min. After centrifugation, 500 L of the supernatant was placed in a 1000 mL volumetric flask and diluted with mobile phase to scale to obtain the sample solution, which was sealed with a sealing

film and stored in a refrigerator at 4 ° C.

The prepared reference substance and sample solution were respectively injected under LC/MS conditions, and the specificity of the method was investigated, as shown in Figure 7, the sample and the standard had the same chromatographic peak in the corresponding position of the chromatogram.

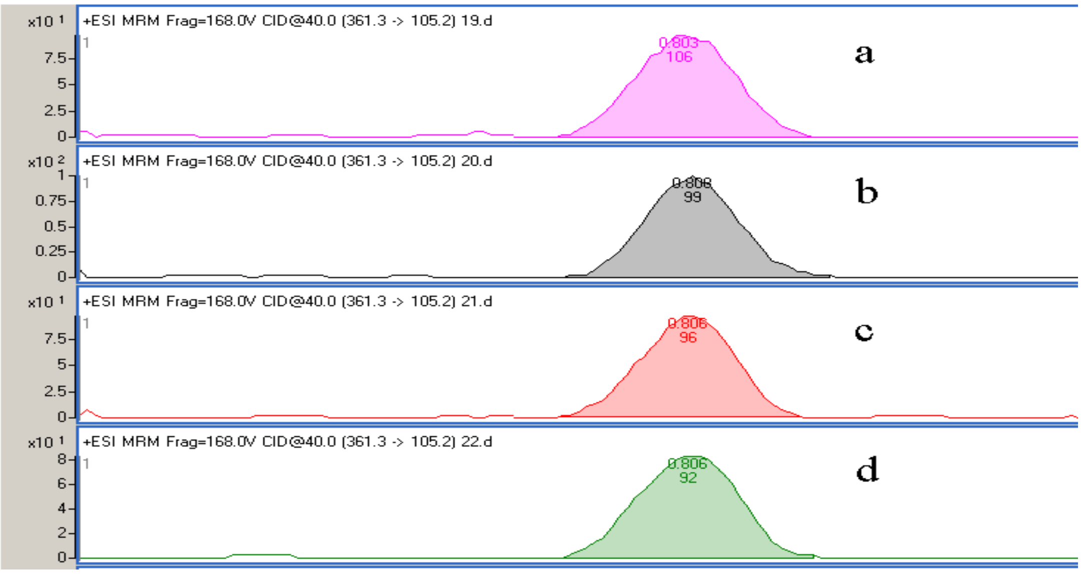

Figure S7. Mass spectrum of lactone armor (a), TPL nanoemulsions (b), TPL nanoemulsion gels (c) and TPL gels (d)

Establishment of standard curves

The sample solutions of 0.1 mL, 1 mL, 2 mL, 3 mL, 4 mL and 5 mL were carefully measured and placed in 10 mL volumetric bottles, and then the volume was fixed to 10 mL with methanol. Standard solutions with concentrations of 10 ng/mL, 100 ng/mL, 200 ng/mL, 300 ng/mL, 400ng/mL and 500ng/mL were prepared. Samples were injected sequentially according to the chromatographic conditions mentioned above. Using triptolide concentration (X) as abscess and peak area (Y) as ordinate, the standard curve equation with a linear range of 10-500 ng /mL was obtained: Y=3.3827x-12.053, r=0.9999. The results showed that triptolide had good linearity in the range of 10-500 ng /mL.

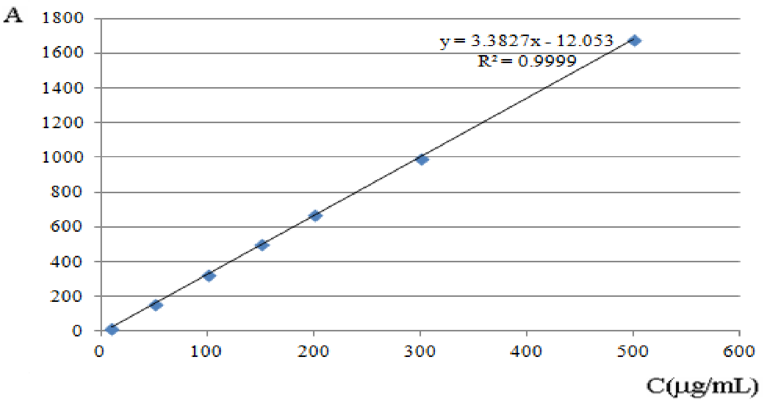

Figure S8. LC/MS standard curve of TPL

Investigation of stability

The standard of triptolide was accurately weighed and prepared into 200 ng/mL standard solution. After preparation of the solution to be tested, the sample was injected and measured at 0, 2, 4, 6, and 8 h. The peak area of triptolide was measured according to LC/MS conditions, and the results were shown in table 1. The RSD value was 0.29%, indicating that the samples were basically stable within 8 hours after treatment.

Table S1. Results of stability test（*n* = 6）

Time	0 h	2 h	4 h	6 h	8 h	$\bar{X}$	RSD %
Peak area	661	662	660	657	659	660	0.29

Experiment of precision

The low, medium and high concentrations of triptolide reference solution were 10 ng/mL, 100 ng/mL and 500 ng/mL, respectively, and the samples were injected at 0, 4, 8, 12 and 24h within the day and at 1D, 2D, 3D and 4D during the day, respectively. The peak area of triptolide was determined according to the chromatographic conditions under one item. The results were shown in Table 2 and 3. The precision RSD values were 4.70%, 1.79%, 0.75%, respectively, and the daytime precision RSD values were 5.55%, 2.46%, 0.45%, respectively. The results showed that the precision of this method was good.

Table S2. Results of intraday precision test（*n*=6）

C/T	0 h	4 h	8 h	12 h	24 h	$\bar{X}$	RSD %
10 ng/mL	18	19	18	17	17	18	4.70
100 ng/mL	323	314	322	325	330	323	1.79
500 ng/mL	1677	1670	1698	1665	1679	1678	0.75

Table S3. Results of Daytime precision test（*n* = 6）

C/T	1 d	2 d	3 d	4 d	$\bar{X}$	RSD %
10 ng/mL	17	18	18	16	17	5.55
100 ng/mL	330	337	321	320	327	2.46
500 ng/mL	1679	1670	1667	1683	1675	0.45

Sample recovery experiment

The low concentration, medium concentration and high concentration of triptolide standard solution were added into the sample solution with known concentration. The sample was injected for determination and the recovery rate was calculated. The recovery results are shown in Table 4.

Table S4. Results of recovery test（*n* = 6）

Sample concentration (ng/mL)	Addition amount (ng/mL)	Measured amount (ng/mL)	Recovery rate (%)	Average recovery rate (%)	RSD%
	5	14.32	95.47		
10	10	20.19	100.95	98.53	2.88
	15	24.87	99.48		
	100	199.20	99.60		
100	200	300.12	100.04	99.76	0.24
	300	398.54	99.64		
	150	350.87	100.35		
200	250	448.96	99.77	100.05	0.29
	350	550.12	100.02		
